# Author Correction: An extended Weight Kernel Density Estimation model forecasts COVID-19 onset risk and identifies spatiotemporal variations of lockdown effects in China

**DOI:** 10.1038/s42003-021-01924-6

**Published:** 2021-03-22

**Authors:** Wenzhong Shi, Chengzhuo Tong, Anshu Zhang, Bin Wang, Zhicheng Shi, Yepeng Yao, Peng Jia

**Affiliations:** 1 Smart Cities Research Institute, Department of Land Surveying and Geo-Informatics, The Hong Kong Polytechnic University, 999077 Hong Kong, China; 2 College of Oceanography and Space Informatics, China University of Petroleum, 266580 Qingdao, China; 3 Research Institute for Smart Cities, School of Architecture and Urban Planning, Shenzhen University, 518060 Shenzhen, China; 4 International Institute of Spatial Lifecourse Epidemiology (ISLE), 999077 Hong Kong, China

**Keywords:** Risk factors, Infectious diseases

Correction to: *Communications Biology* 10.1038/s42003-021-01677-2, published online 25 January 2021.

In the original version of this Article, Figs. 2, 3a, and 4 were missing a map element. These errors have been corrected in the HTML and PDF versions of the paper, and do not affect the scientific content of the figures.

















The original version of the Article included references to “all 347 Chinese cities” or “all Chinese cities,” in the Abstract, the fifth paragraph of the Introduction, the fifth paragraph of the Results, and the first paragraph of the Discussion. In these instances the word “all” has now been omitted in the PDF and HTML versions of the Article.

In the original version of the Article, the fifth paragraph of the Results stated “In all 347 cities of China…” The text “of China” has now been omitted in the HTML and PDF versions of the paper.

In the original version of the Article, the fifth paragraph of the Introduction stated “The 347 Chinese cities include all prefecture-level cities...” The text has now been amended to include “The 347 Chinese cities include all prefecture-level cities except for Sansha City which is free of COVID-19 infections…” in the HTML and PDF versions of the paper.

